# Progress in non-invasive neuromodulation based on consciousness-related neural circuits: a narrative review

**DOI:** 10.3389/fneur.2026.1770928

**Published:** 2026-04-01

**Authors:** Yichen Wang, Yiyi Zhai, Zhiyao Zheng, Nan Wang, Xiaoke Chai, Haodong Niu, Yitong Jia, Sipeng Zhu, Yuhan Shang, Kefei Wan, Tianqing Cao, Qiheng He, Tan Zhang, Hancheng Qiu, Yi Yang

**Affiliations:** 1 Beijing Tiantan Hospital, Capital Medical University, Beijing, China; 2 Department of Neurosurgery, Beijing Tiantan Hospital, Capital Medical University, Beijing, China; 3 China National Clinical Research Center for Neurological Diseases, Beijing, China; 4 Department of Neurosurgery, Peking Union Medical College Hospital, Chinese Academy of Medical Sciences and Peking Union Medical College, Beijing, China; 5 Brain Computer Interface Transitional Research Center, Beijing Tiantan Hospital, Capital Medical University, Beijing, China; 6 Department of Neurosurgery, Aviation General Hospital, Beijing, China; 7 Department of Neurosurgery, The Second Affiliated Hospital of Soochow University, Suzhou, China; 8 National Research Center for Rehabilitation Technical Aids, Beijing, China; 9 Chinese Institute for Brain Research, Beijing, China; 10 Beijing Institute of Brain Disorders, Beijing, China

**Keywords:** consciousness-related neural circuits, cortico-thalamocortical circuit, disorders of consciousness, frontoparietal network, non-invasive neuromodulation

## Abstract

Disorders of Consciousness (DOC) are characterized by abnormal function or disrupted connectivity of consciousness-related neural circuits, mainly presenting as Vegetative State/Unresponsive Wakefulness Syndrome (VS/UWS) and Minimally Conscious State (MCS), which impose a heavy burden on patients’ families and society. Non-Invasive Brain Stimulation (NIBS) has emerged as a core research direction for DOC treatment due to its non-invasiveness, ease of operation, and favorable safety profile. Based on the classification of consciousness-related neural circuits, this review systematically summarizes the research progress of central and peripheral non-invasive neuromodulation techniques, including their potential regulatory mechanisms on core circuits (such as the frontoparietal network, cortico-thalamocortical circuit, and ascending reticular activating system), clinical evidence, and synergistic effects of combined therapies. Studies have shown that techniques like Transcranial Magnetic Stimulation (TMS) and Transcranial Direct Current Stimulation (tDCS) targeting the frontoparietal network, Low-Intensity Transcranial Focused Ultrasound (LITUS, also referred to as Transcranial Focused Ultrasound [TUS]/transcranial Focused Ultrasound [tFUS] in the field) and Temporal Interference (TI) regulating the cortico-thalamocortical circuit, and Median Nerve Stimulation (MNS) activating the ascending reticular activating system have demonstrated certain efficacy in improving consciousness in MCS patients, while the evidence for efficacy in VS/UWS patients remains weak due to small sample sizes, lack of control groups and insufficient statistical power. Combined therapies such as TMS + MNS and Transcranial Focused Ultrasound LITUS+TMS exhibit significantly superior synergistic effects compared to monotherapies. By horizontally comparing the advantages and limitations of various techniques, this review proposes personalized treatment recommendations based on the characteristics of neural circuit damage. It also points out that future research should optimize stimulation parameters, clarify the specificity of circuit regulation, and verify long-term efficacy through large-sample randomized controlled trials (RCTs), aiming to provide a reference for the standardized and precise application of NIBS in DOC treatment.

## 1 Introduction

Disorders of Consciousness (DOC) refer to a state of severe loss of conscious perception caused by severe brain injury (1). Their core pathological mechanisms are thought to involve weakened excitatory synaptic activity and disrupted connectivity of consciousness-related neural circuits (2). Consciousness-related neural circuits mainly include the frontoparietal network, cortico-thalamocortical circuit, ascending reticular activating system, limbic system, and silent system (3). Damage to different circuits may lead to varying degrees of impairment in arousal and awareness functions. Key nodes in these circuits have become stimulation targets for non-invasive neuromodulation, holding promise for promoting wakefulness in patients (3).

Currently, DOC is mainly classified into three categories: coma, Vegetative State/Unresponsive Wakefulness Syndrome (VS/UWS), and Minimally Conscious State (MCS) (4). China sees 50,000 to 100,000 new cases annually, creating an urgent demand for effective treatments (1). Pharmacological treatments are mostly empirical, with insufficient evidence-based medical support except for amantadine (4). In contrast, non-invasive neuromodulation techniques, by targeting abnormal neural circuits, have shown significant potential in improving consciousness and have become an important research direction for DOC treatment (5).

Non-invasive neuromodulation techniques are categorized into central (directly acting on the cerebral cortex or deep nuclei) and peripheral (indirectly regulating through peripheral nerve-central pathways) based on stimulation sites, both aiming to repair or regulate the function of consciousness-related neural circuits (6). Focusing on the classification of core consciousness-related neural circuits, this review systematically summarizes the potential mechanisms of action, clinical evidence, and application prospects of various non-invasive neuromodulation techniques, providing theoretical support for precise treatment. The targeting of key nodes of the neural circuit by different noninvasive neuromodulation modalities is summarized in Table 1.

**Table 1:** Summary treatment, regions and limitations of noninvasive neuromodulation techniques.

Category	Name of the means	Treatment	Targeting brain regions	Limitations	Evidence strength and reason	Citations
Central neuromodulation technology	Transcranial Magnetic Stimulation (TMS)	Brain cells are activated by electromagnetic induction	Pallium (dorsolateral prefrontal cortex, angular gyrus, etc.)	The efficacy against VS is weak, and the evidence for stimulation targets such as angular gyrus is insufficient	Weak for VS (*n* < 30 in most studies, no multi-center RCTs); Moderate for MCS (controlled trials with statistical significance)	(21, 23, 26)
	Transcranial direct current electromagnetic (tDCS)	Direct current stimulation increases cortical excitability	Pallium (dorsolateral prefrontal cortex, precuneus)	The efficacy of VS is weak, and the multi-target regimen needs to be verified	Weak for VS (no significant improvement in controlled trials); Moderate for MCS (large-sample RCTs with significant outcomes)	(27–29)
	Transcranial alternating current stimulation (tACS)	Alternating current synchronizes oscillating frequencies inside and outside the brain	Pallium (dorsolateral prefrontal lobe, frontopolar lobe)	There are few direct studies on DOC treatment, and there is no clear awakening effect	Very weak (only 2 small-sample studies, no RCTs for DOC)	(30, 31)
	Time-domain interference (TI)	The difference in alternating current frequency forms an envelope electric field to regulate specific brain regions	Deep brain region (thalamus)	There are few studies applied to docs, and the mechanism needs to be further clarified	Very weak (only animal and healthy human studies, no clinical data for DOC)	(39, 41, 44)
	Low-intensity transcranial ultrasound (LITUS)	Low-intensity ultrasound regulates neurons and brain networks	Deep brain region (thalamus)	The sample size is small, the spatial accuracy is limited, and the long-term efficacy is unknown	Weak (*n* < 15 in total clinical studies, single-center trials only)	(40, 42, 43)
Peripheral neuromodulation techniques	Median Nerve Electrical Stimulation (MNS)	The ascending reticular system and cerebral cortex are activated by electrode electric fields	Deep nucleus + cerebral cortex	The effect on the right side is better than that on the left side, and the long-term efficacy has not been verified	Moderate (large-sample RCTs, significant outcomes for both MCS and mild VS)	(5, 51)
	Transcutaneous Electrical Vagus Nerve Stimulation (taVNS)	Stimulates the vagus nerve to regulate the limbic system	Limbic system + ascending reticular activating system	The sample size is small, the scope of clinical application is limited, and the research on the mechanism of action has not yet been clarified	Very weak (*n* < 20 in total clinical studies, case reports and small pilot trials)	(48, 53)
	Electrical Stimulation of the Trigeminal Nerve (TNS)	Stimulates the trigeminal nerve to regulate brain networks and improve cerebral blood flow	Limbic system + ascending reticular activating system	The number of experiments is small, the research on DOC started late (2018 for first case report) and there is still a lot of room for future development	Weak (only 1 RCT with *n* = 60, short follow-up time)	(57–59)

DOC, Disorders of Consciousness; VS/UWS, Vegetative State/Unresponsive Wakefulness Syndrome; MCS, Minimally Conscious State; CRS-R, Coma Recovery Scale-Revised; GCS, Glasgow Coma Scale; DLPFC, Dorsolateral Prefrontal Cortex; AG, Angular Gyrus; PCUN, Precuneus; Thal, Thalamus; ARAS, Ascending Reticular Activating System; LS, Limbic System; NE, Norepinephrine; DA, Dopamine; TBI, Traumatic Brain Injury.

## 2 Non-invasive neuromodulation targeting the frontoparietal network: cortico-cortical circuit

The frontoparietal network serves as the core circuit for conscious awareness and cognitive control (7). Reduced functional connectivity and neuronal excitability within this network are key pathological features of DOC (8). Core targets of this network include the Dorsolateral Prefrontal Cortex (DLPFC), angular gyrus, and precuneus. Non-invasive neuromodulation techniques such as TMS and tDCS applied to these targets may regulate neuronal excitability, enhance cortico-cortical circuit connectivity, repair impaired arousal and awareness functions in DOC patients, thereby achieving potential clinical improvement (8).

The frontoparietal network is formed by neural fiber connections between key brain regions of the prefrontal and parietal lobes, responsible for conscious awareness and cognitive control (9). The DLPFC maintains extensive connections with multiple cortical regions, governing cognitive control and information integration, while also having close links with subcortical structures (e.g., thalamus, basal ganglia) and participating in goal maintenance and multi-task processing (10). The angular gyrus connects extensively with the Prefrontal Cortex (PFC), involved in working memory maintenance/updating, multi-sensory information integration, and attention regulation (11). Located on the medial surface of the parietal lobe, the precuneus forms the Default Mode Network (DMN) through tight connections with the medial Prefrontal Cortex (mPFC), participating in self-awareness, self-reflection, attention regulation, and maintenance of conscious states (12).

### 2.1 DLPFC—cortex

The DLPFC, as a stimulation target, communicates with surrounding cortices through numerous neural fibers. Current stimulation methods targeting the DLPFC mainly include TMS, tDCS, and tACS (10).

Transcranial Magnetic Stimulation (TMS): It may regulate the action potentials of neurons in the DLPFC and frontoparietal cortex. High-frequency TMS significantly enhances cortical excitability, neural metabolism, and cerebral blood flow, which may reverse the inhibitory neural state in DOC patients (13, 14). Repetitive TMS (rTMS) can also produce sustained effects through the carry-over effect (15), a phenomenon where the neuroplastic changes induced by stimulation persist beyond the stimulation period, which may further enhancing functional connectivity within the prefrontal cortical circuit by promoting long-term potentiation (LTP)-like synaptic modifications (15).

Transcranial Direct Current Stimulation (tDCS): It directly regulates the neuronal excitability of the frontoparietal network at the DLPFC by delivering weak direct current (1 mA–2 mA) (16), depolarizing cortical neurons and enhancing Motor Evoked Potentials (MEP). Additionally, tDCS induces changes in cerebral blood flow and neurotransmitter balance, which may exert significant improvements in frontoparietal brain network connectivity and conscious states (17–19).

Transcranial Alternating Current Stimulation (tACS): It applies sinusoidal alternating current of specific frequencies through scalp electrodes, entraining the brain’s intrinsic oscillations (e.g., θ, α, γ bands) to the DLPFC. This may synchronize the activity of neurons near the target with the stimulation frequency, thereby potentially influencing brain rhythms in the prefrontal cortical area, promoting the activation of consciousness-related neural circuits, and improving brain function (16, 20).

#### 2.1.1 Clinical evidence for TMS stimulation of the DLPFC

Current research confirms that the DLPFC is the most definitive and effective target for TMS (21). Early TMS studies in 2013 primarily targeted the Primary Motor Cortex (M1) and DLPFC (22). Ge et al. (23) conducted a controlled trial targeting the DLPFC, involving 32 DOC patients (15 in the rTMS group and 17 in the control group). The rTMS protocol used 10-Hz stimulation, with intensity set at 90% of the resting motor threshold (RMT), administered once daily for 10 consecutive days (23). After treatment, the median Coma Recovery Scale-Revised (CRS-R) score increased by 3 points in the rTMS group, significantly higher than the 1-point increase in the control group (*p* < 0.001), with a consciousness conversion rate of 86.7% (much higher than 29.4% in the control group, *p* = 0.0016). Compared with the earliest effects of TMS applied to the M1 cortex by Mangaotti in 2013, who used 5-Hz rTMS at 80% RMT for 5 consecutive days (22), the results were more definitive—obvious improvements in patients’ conscious states were observed, including increased spontaneous arousal, improved visual pursuit and object localization, and enhanced motor responses to external stimuli, confirming that the DLPFC is a superior stimulation target.

Recent studies have achieved enhanced standardization and efficacy. In 2022, Fan et al. (24) randomly divided 40 DOC patients into an active rTMS group and a sham stimulation control group. The active group received 20-Hz rTMS to the left DLPFC (intensity: 100% resting motor threshold), with a total of 3,000 pulses per session, administered once daily for 14 consecutive days (24). The post-treatment consciousness improvement score (8.45 ± 3.55) was significantly higher than that of the control group (6.25 ± 1.29). Xia et al. (25) applied 10-Hz rTMS to the DLPFC of 16 DOC patients (5 MCS, 11 VS). The stimulation targeted the left DLPFC, with intensity at 90% RMT, 2000 pulses per session, once daily for 10 days (25). All 5 MCS patients showed increased CRS-R scores (average increase of 2.4 points), and 3 VS patients emerged from the vegetative state; this increase in CRS-R scores for MCS patients was numerically improved but not statistically significant due to the small sample size (*n* = 5).

In 2025, Liu et al. 26conducted a latest controlled trial involving 48 DOC patients. The protocol used 15-Hz rTMS at 100% RMT, targeting the left DLPFC, with 3,000 pulses per session, once daily for 12 consecutive days (26). Fifty percent (12/24) of patients in the rTMS group showed progression in consciousness classification, significantly higher than 20.8% (5/24) in the control group (95% CI: 3.40–54.9%), with an adjusted Relative Risk (RR) = 3.06 (95% CI: 1.54–6.09, *p* = 0.001). An 8-week follow-up revealed that the consciousness improvement rate in the rTMS group (54.2%) remained significantly higher than that in the control group (29.2%), with an adjusted RR = 2.37 (95% CI: 1.36–4.14, *p* = 0.002).

#### 2.1.2 Clinical evidence for tDCS stimulation of the DLPFC

Research on tDCS stimulation of the DLPFC is relatively sufficient. In the first double-blind randomized controlled trial conducted by Thibaut et al. (27), 55 DOC patients (including UWS and MCS) received 20-min, 2 mA tDCS to the left DLPFC. While the overall treatment effect was not significant, the subgroup of 30 MCS patients showed significant improvements.

In a stimulation experiment by Zhang et al. (28), 26 DOC patients were divided into a real stimulation group (5 VS/8 MCS) and a sham stimulation group (6 VS/7 MCS). The real stimulation group received anodal tDCS to the left DLPFC (20 sessions, 10 consecutive working days, twice daily; 1 mA intensity for patients with left craniotomy). The results showed that the total CRS-R score of MCS patients in the real stimulation group significantly increased after treatment (*F* = 72.54, *p* < 0.0005), with statistically significant improvements in all six sub-items: auditory, visual, motor, language, communication, and arousal. No significant differences in CRS-R scores were observed before and after treatment in VS patients (real stimulation group) and the sham stimulation group (regardless of VS/MCS) (*p* > 0.05).

A latest meta-analysis by Barra et al. (29) integrated data from 8 studies involving 131 DOC patients through multi-center RCT data and LASSO logistic regression model analysis, confirming the wake-promoting efficacy of tDCS stimulation of the DLPFC in DOC patients and identifying MCS as the main beneficiary population.

#### 2.1.3 Clinical evidence for tACS stimulation of the DLPFC

Early tACS studies in 2017 were mostly conducted on healthy individuals. Violante et al. (30) applied 10-Hz in-phase tACS (frontopolar-Fp1/parietal-P3) to healthy subjects, resulting in a statistically significant 15–20% improvement in working memory accuracy (*p* < 0.05) and a statistically significant 40% increase in frontoparietal θ wave (4 Hz-7 Hz) synchronization (*p* < 0.01); anti-phase stimulation led to a statistically significant 10% decrease in accuracy (*p* < 0.05), suggesting that in-phase tACS may restore frontoparietal network communication.

In recent years, tACS has been formally applied in treatment research for DOC patients. Naro et al. (31) conducted a controlled trial (randomized crossover design, each patient received tACS and transcranial Random Noise Stimulation (tRNS) sequentially) of γ-band (35 Hz-140 Hz) tACS on the right DLPFC and frontopolar cortex in 20 chronic DOC patients (10 VS/UWS, 10 MCS). The results showed that:

MCS patients had a significant increase in the relative power of θ waves (4 Hz–7 Hz) and γ waves (35 Hz–140 Hz) in the frontoparietal region after tACS stimulation (*p* < 0.05), while VS patients only showed an increase in γ wave power. All MCS patients and 30% (3/10) of VS/UWS patients exhibited a significant improvement in frontoparietal network functional coherence (reflecting inter-brain region information transmission) after tACS, mainly in the θ and γ bands (*p* < 0.05). This indicates that tACS may repair cortico-cortical circuits and improve frontoparietal network connectivity.

### 2.2 Precuneus—cortex

The precuneus (PCUN) is a core brain region for conscious processing, involved in memory retrieval, spatial cognition, and frontoparietal network regulation (32). Its injury probability in DOC patients is lower than that of the DLPFC, and its metabolic level is closely related to the prognosis of consciousness recovery—making it one of the potential optimal targets for DOC neuromodulation (12). Currently, the main stimulation method for the precuneus is tDCS (12).

#### 2.2.1 Clinical evidence for tDCS stimulation of the PCUN

There are relatively few studies targeting the precuneus with tDCS, and the sample sizes are small. However, existing studies have shown significant and clear improvements in patients’ conscious levels:

Huang et al. (33) found that although precuneus tDCS can improve conscious levels, its effect is weaker than that of the DLPFC target. In a latest study by Guo et al. (34), 11 DOC patients received high-definition tDCS (HD-tDCS) with anodal stimulation on the precuneus. T0 represents the baseline before treatment, T1 represents 1 day after treatment, T2 represents 7 days after treatment, and T3 represents 14 days after treatment (34). The total CRS-R score gradually increased over time after treatment: it was significantly higher at T2 (7 days) than at T0 (*p* < 0.05), and even more significantly higher at T3 (14 days) (T0: 7.2 ± 2.1 points → T3: 9.8 ± 2.5 points, *p* < 0.01). All 6 MCS patients showed improvements, with an average increase of 5.2 points in CRS-R scores.

In the study by Guo et al. (34), electrophysiological indicators revealed: The short-range coherence of central-parietal regions at T3 was significantly lower than that at T0 (*p* < 0.05), suggesting that excessive synchronization of the local neuronal network in the precuneus was inhibited. Starting from T2 (7 days), the long-range coherence between frontal hemispheres and central hemispheres significantly decreased (*p* < 0.05), indicating an improvement in global cross-regional network integration function.

### 2.3 Angular gyrus—cortex

The angular gyrus (AG) has bidirectional neural projections with the prefrontal cortex and extensive neural connections with other regions in the parietal lobe (e.g., postcentral gyrus) (12). Currently, TMS may activate the internal neural network of the frontoparietal lobe by stimulating the angular gyrus, regulating functions such as attention concentration, spatial judgment, and cognitive control in DOC patients (35).

#### 2.3.1 Clinical evidence for TMS stimulation of the AG

As a potential target, the angular gyrus has achieved positive results in clinical practice. Legostaeva et al. (35) applied 20-Hz rTMS to the angular gyrus (parieto-occipital junction) of 22 MCS patients, and 19 of them showed an increase in total CRS-R scores and improved conscious levels. However, the number of studies and sample sizes targeting the angular gyrus still need further expansion and exploration (35).

## 3 Non-invasive neuromodulation targeting the cortico-thalamocortical circuit

The bidirectional closed-loop cortico-thalamocortical circuit is also a key pathway for wake-promoting in DOC, responsible for maintaining arousal and screening/integrating information (3). Its key targets include the thalamus, prefrontal cortex, and parietal cortex. Abnormal connections between the thalamus and the prefrontal/parietal cortex are core pathological links in DOC (36). Techniques targeting this circuit are mainly characterized by deep thalamic regulation, such as LITUS and TI.

Located in the deep central region of the brain, the thalamus acts as a cortical gateway: it receives peripheral sensory signals and projects them to the cortex, which in turn feedback-regulates thalamic activity—achieving information screening, synchronous integration, and maintaining the foundation of arousal and awareness.

The prefrontal cortex and parietal cortex receive sensory projections from the thalamus and feedback-regulate the thalamus; however, the regulatory effect of stimulating most cortical targets on the thalamus is mostly indirect and unclear. Emerging evidence has demonstrated that LITUS targeting the primary somatosensory cortex (S1) may modulate the cortico-thalamocortical (CTC) pathway by selectively activating excitatory neurons, providing a novel indirect approach for thalamic regulation (37).

### 3.1 Thalamus—cortex

As a deep brain region and a core node in the pathogenesis of DOC, the thalamus can currently be directly regulated by non-invasive techniques mainly including Low-Intensity Focused Transcranial Ultrasound (LITUS) and Temporal Interference (TI).

LITUS: With high spatial resolution and good penetration depth, it can precisely target the thalamus. At the circuit level, LITUS may activate thalamic neurons, enhances excitability, strengthens bidirectional connections between the thalamus and the prefrontal/parietal cortex, potentially restoring thalamocortical signal transmission, and improves arousal levels. At the molecular level, it may inhibit M1 polarization of microglia, reduces neuroinflammatory responses, avoids the accumulation of neurotoxic substances, and improves the neuronal microenvironment. At the cellular level, it may activate autophagic pathways, clear abnormal protein aggregates, and repair damaged thalamic neurons (38, 39).

Temporal Interference (TI): It generates low-frequency envelope electric fields in deep brain regions through two groups of high-frequency alternating currents with slight frequency differences (40), which may precisely regulate neuronal activity in the cortico-thalamocortical circuit (enhancing or weakening network synchronization). It only acts on the target area without activating the superficial cortex, ensuring high safety (40, 41).

#### 3.1.1 Clinical evidence for LITUS targeting the thalamus

Monti et al. (42) first focused LITUS on the thalamus and reported that a DOC patient showed improvements in both the Glasgow Coma Scale (GCS) and CRS-R scores after thalamic-focused ultrasound stimulation—confirming that LITUS can upregulate the excitability of the cortico-striato-pallido-thalamo-cortical pathway.

Cain et al. (43) included 3 DOC patients in a double-blind, sham-stimulation controlled trial using the same parameters as Monti. The parameters included ultrasound frequency of 650 kHz, acoustic intensity of 0.3 W/cm^2^, and stimulation duration of 30 min per session, administered once daily for 5 consecutive days (42, 43). Two patients showed clinically significant improvements in CRS-R scores (increase ≥2 points) after each ultrasound stimulation, accompanied by new consciousness-related behaviors (e.g., active visual tracking, pain stimulus localization). Even in patients with chronic DOC (disease duration >1 year), unprecedented behaviors (e.g., attempting to express needs through gestures) were observed, but the long-term effect was poor and required repeated stimulation to maintain efficacy.

In 2022, Monti’s team expanded the trial size 42, including 11 acute DOC patients (2 comatose, 3 VS/UWS, 6 MCS). The protocol used 650 kHz LITUS at 0.3 W/cm^2^, 30 min per session, once daily for 10 consecutive days (44). After treatment, patients showed significant improvements in behavioral performance, and changes in thalamic connectivity were positively correlated with improvements in the CRS-R index after LITUS stimulation. The median CRS-R index at 1 week after treatment (12.3) was significantly higher than the baseline (8.7) (*p* = 0.014), and the results of the raw CRS-R total score were consistent (p = 0.014). Among the 11 patients, 4 achieved an upgrade in consciousness state (1 comatose → VS, 1 VS → MCS−, 2 VS → MCS+). These findings suggest that LITUS can precisely target the thalamus and its deep circuits, providing a new approach for chronic DOC treatment.

#### 3.1.2 Clinical evidence for TI targeting the thalamus

TI is highly selective, can directly regulate the thalamus, exhibits strong deep targeting, and a high degree of safety. Grossman’s team (40) first verified the effect through animal experiments: TI stimulation (high-frequency difference of 10-Hz vs. direct application of 10-Hz current) was applied to the hippocampus of mice. c-fos detection showed that the activation rate of the hippocampal dentate gyrus reached 53.12% ± 14.5%, which was close to the effect of direct application of 10-Hz current (difference of approximately 10%), but the superficial cortex was not activated. Meanwhile, it was observed that hippocampal nerve cells under TI stimulation could fire action potentials following the envelope electric field (short spike bursts, burst interval <15 ms) without excessive excitation/inhibition—first confirming the activation effect of TI on neurons in deep brain regions.

Based on animal experiments, Violante et al. (41) verified the characteristics of TI’s action on the human brain: (1) verifying the precise targeting of the human hippocampus through TI stimulation of cadavers; (2) applying 5-Hz TI stimulation to 20 healthy individuals in two groups (1:1 and 1:3), with stimulation duration of 20 min per session, administered once daily for 5 consecutive days (41). The study confirmed the changes in stimulation focus under different current ratios and significant stimulation effects; (3) conducting a TI stimulation vs. sham stimulation controlled trial on 21 healthy individuals. The results showed that the TI group had better memory correctness and stability, and TI stimulation reduced the functional connectivity (FC) between the hippocampus and the Default Mode Network (DMN). In the hippocampal region affected by the maximum envelope modulation amplitude, the relative connectivity was higher—confirming that the regulatory effect of TI on deep human brain regions is related to the intensity of the envelope electric field (theta burst TI, tTIS).

Liu et al. (45) conducted a combined animal and human study: TI stimulation was applied to 2 rhesus monkeys, with stimulation intensity of 2 mA, duration of 30 min per session, once daily for 7 consecutive days in the animal model (45). The study found that under 2 mA stimulation, the actual transcranial induced power in the deeper midline region exceeded the threshold for traditional TMS to regulate neuronal firing time (0.2 V/m), having the potential to modulate neuronal activity. For human participants with movement disorders, the protocol used 2 mA TI stimulation, 20 min per session, twice weekly for 4 weeks (45). Subsequently, TI and tACS were applied to patients with movement disorders for comparative stimulation (bilateral substantia nigra), and the patients’ motor scores were significantly improved, with TI power reaching 0.125 V/m-0.22 V/m—further confirming that neurons in deep human brain nuclei can achieve effective firing intensity through TI, providing feasibility evidence for DOC treatment.

## 4 Non-invasive neuromodulation targeting the ascending reticular activating system and limbic system

The Ascending Reticular Activating System (ARAS) is the power core of conscious arousal, and the Limbic System (LS) is the regulatory hub for integrating emotions, memories, and consciousness (3, 46, 47). Non-invasive neuromodulation stimulates targets on these circuits to activate arousal pathways, regulate neurotransmitter balance, repair network connectivity, and improve the arousal level and awareness integration ability of DOC patients (3, 48).

### 4.1 Core circuits and targets

ARAS is a diffuse neural network running through the brainstem, whose core function is to maintain the awake state of the brain and provide the foundation for awareness. Its damage directly leads to loss of arousal (3, 46). Key targets include midbrain reticular formation, locus coeruleus and raphe nucleus. Midbrain reticular formation integrates peripheral sensory signals and projects them to the thalamus to initiate arousal. Stimulation targeting locus coeruleus may promote the release of norepinephrine, regulating cortical excitability and attention. Raphe nucleus may release serotonin, regulating the sleep–wake cycle (3, 46).

Limbic System (LS) is a subcortical network surrounding the brainstem, whose core function is to integrate emotions, memories, and sensory information. It interacts with ARAS and the frontoparietal loop to realize the transformation from arousal to awareness (49). Key targets include hypothalamus, amygdala and hippocampus. Stimulation targeting hypothalamus may promote the release of orexin to maintain arousal. Amygdala interacts with the thalamus to assist arousal. Hippocampus is involved in memory formation/retrieval and multi-sensory information integration, helping the recurrence of awareness-related behaviors (47).

### 4.2 Representative techniques and clinical evidence

#### 4.2.1 Median nerve stimulation that targets the ascending reticular activating system and limbic system

Median nerve stimulation (MNS) activates the median nerve at the wrist through electrodes. Peripheral sensory signals are transmitted upward through the spinal cord, activating the brainstem ARAS and projecting to deep brain regions (e.g., hypothalamus). It works by: (1) activating the locus coeruleus of ARAS to release norepinephrine (NE) and the raphe nucleus to release serotonin (5-HT), enhancing hypothalamic cerebral blood flow; (2) upregulating the expression of orexin-A and its receptor (OX1R), enhancing glutamatergic neurotransmission, and activating NMDA receptors to promote arousal; (3) increasing the release of brain-derived neurotrophic factor and promoting synaptic connections to maintain brain arousal function (5, 48, 50).

Research on MNS began in 1999: Cooper et al. (51) performed right MNS on 35 comatose patients. The parameters included stimulation intensity of 20 mA, pulse width of 300 μs, frequency of 40 Hz, and duration of 8 h per day for 2 weeks (51). The study found that the treatment group had higher GCS scores and shorter ICU stay time. Among 10 traumatic brain injury (TBI) patients with GCS scores of 4–8, 6 had shortened average wakefulness time after 2 weeks of right MNS treatment; Single-Photon Emission Computed Tomography (SPECT) showed increased cerebral blood flow perfusion in the bilateral cortex, basal ganglia, and thalamus.

In 2023, Wu et al. (52) conducted the largest randomized controlled trial to date, involving 329 patients (167 in the MNS group, 162 in the control group). The MNS group received 8 h of daily stimulation for 2 weeks (parameters: 20 mA, 300 μs, 40 Hz). After 6 months: (1) The proportion of consciousness recovery in the MNS group (71.95%) was significantly higher than that in the control group (55.97%) (*p* < 0.001); (2) MNS significantly increased GCS scores (MD = 1.49–2.35, *p* < 0.01), EEG scores (MD = 1.61, *p* < 0.001), and cerebral blood flow velocity (VM MD = 4.23, *p* < 0.05; VS MD = 10.51, *p* < 0.01); (3) It reduced Disability Rating Scale (DRS) scores (MD = −1.77, *p* < 0.05) and shortened ICU stay time (MD = −2.02, *p* < 0.05); (4) It was effective for DOC of different etiologies (e.g., TBI, hypertensive intracerebral hemorrhage [HICH]). MNS is simple to operate, safe, applicable to a wide range of etiologies, and has few adverse reactions (52).

#### 4.2.2 Transcutaneous auricular vagus nerve stimulation that targets the ascending reticular activating system and limbic system

Transcutaneous auricular vagus nerve stimulation (taVNS) stimulates the auricular branch of the vagus nerve to transmit signals to the nucleus tractus solitarius, which then projects upward to the ARAS and limbic system circuits. It works by: (1) promoting the locus coeruleus neurons and dorsal raphe nucleus to release NE and 5-HT; (2) regulating the release of acetylcholine in the basal ganglia to participate in arousal and alertness responses; (3) downregulating inflammation, regulating pain, and improving mood/cognitive function—indirectly improving consciousness state (48).

Research on taVNS started relatively late—taVNS has been widely used in the clinical treatment of depression and epilepsy since the 2000s (53, 54), while its application in DOC treatment was first reported in 2017 (53) and thus remains in the exploratory stage with limited clinical data. In 2017, Yu et al. (53) reported the world’s first case of taVNS in DOC treatment: a 73-year-old female VS patient received 4 weeks of taVNS (stimulating bilateral concha, intensity 4 mA-6 mA), with her CRS-R score increasing from 6 to 13 points (upgrading to MCS) and new motor functions (e.g., limb movement, swallowing, vocalization-related actions). Post-treatment functional Magnetic Resonance Imaging (fMRI) showed enhanced functional connectivity between the posterior cingulate gyrus and multiple brain regions.

In the past 5 years (2020–2025), the sample size of taVNS studies for DOC has increased: Yifei et al. (55) randomly divided 12 DOC patients into taVNS and transcutaneous non-vagal nerve stimulation (tnVNS) groups. After 4 weeks of treatment: (1) The frontoparietal functional connectivity in the taVNS group was significantly enhanced (*p* < 0.05), with a greater enhancement amplitude in the MCS subgroup than in the VS subgroup; (2) No such change was observed in the tnVNS group; (3) The beta wave in the MCS subgroup of the taVNS group increased (*p* < 0.05), indicating improved cortical excitability (consistent with the electrophysiological characteristics of consciousness developing from minimal state to higher-level integration). The CRS-R score increase in the taVNS group (average 3.2 points) was statistically significant compared with the tnVNS group (average 0.8 points, *p* < 0.05). taVNS can regulate neurotransmitter balance in multiple circuits, is non-invasive and easy to operate, and requires more in-depth and precise clinical research (54).

#### 4.2.3 Trigeminal nerve stimulation that targets the ascending reticular activating system and limbic system

Trigeminal nerve stimulation (TNS) activates the locus coeruleus of ARAS, paraventricular nucleus of the hypothalamus, limbic system, and substantia nigra pars compacta by stimulating Tac1 + and Piezo2 neurons in the trigeminal ganglion (TG). It works by: (1) enhancing the release of NE and dopamine (DA) to improve the sensitivity of the visual receptive field and motor function; (2) activating the rostral ventrolateral medulla vascular regulatory center to increase cerebral blood flow (providing a good neuronal microenvironment); (3) exerting neuroprotective effects to reduce secondary brain damage (56, 57).

Fan et al. first proposed TNS (58) for wake-promoting treatment of prolonged DOC (pDOC) patients in 2018, and reported the first successful wake-promoting case in Brain Stimulation in 2019.

In 2023, the team (59) conducted a randomized double-blind sham-controlled study involving 60 pDOC patients (30 in the TNS group, 30 in the sham stimulation group) with a 4-week treatment cycle. The parameters included stimulation intensity of 0.2 mA, pulse width of 200 μs, frequency of 40 Hz, and duration of 60 min per day (59). The results showed that: (1) The GCS and CRS-R scores in the TNS group were more significantly improved after 4 weeks of treatment and 12 weeks of follow-up; (2) After 12 weeks of follow-up, about 50% of patients in the TNS group had improved conscious levels, compared with only 22% in the sham stimulation group.

**Figure 1:**
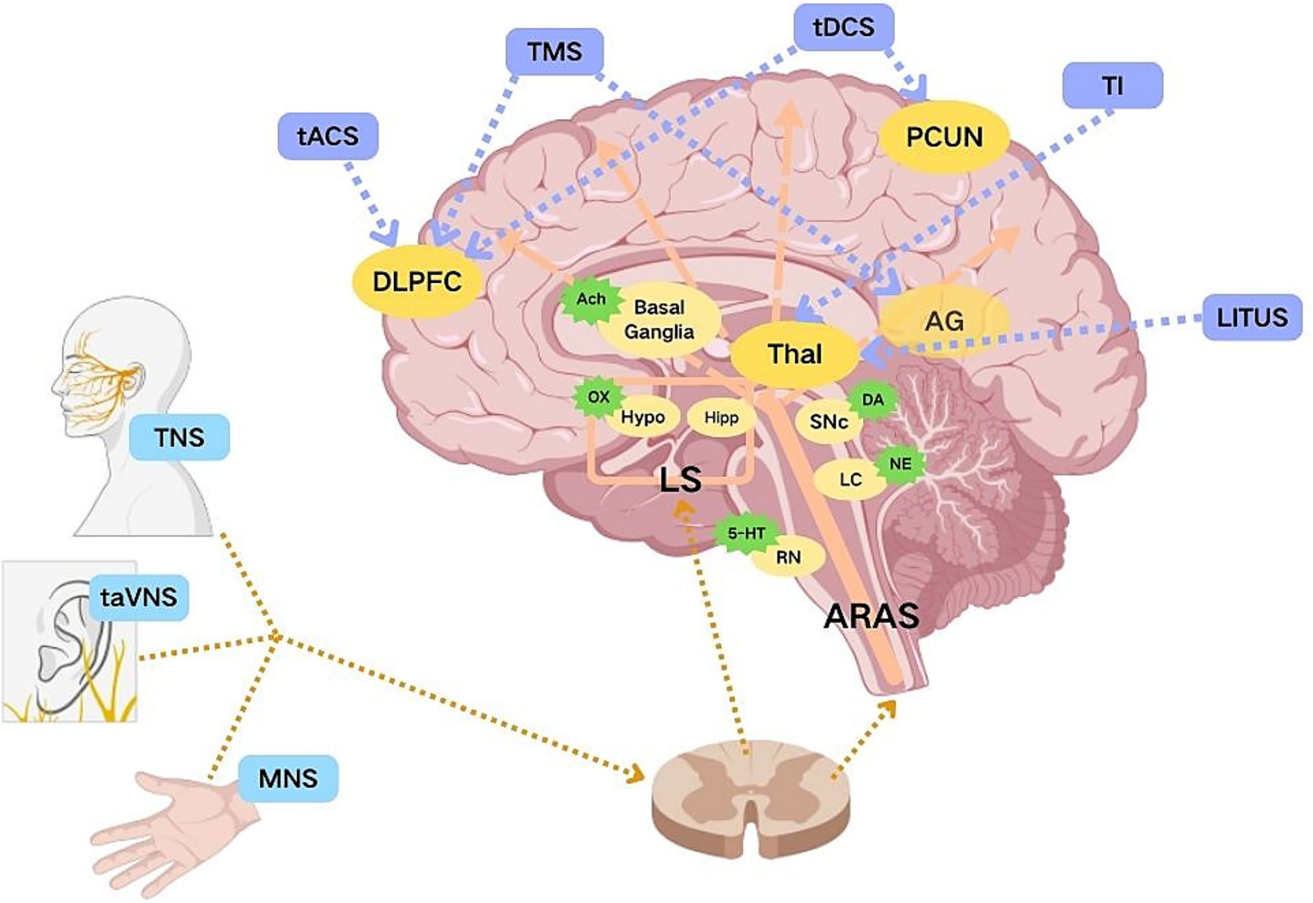
Targets of non-invasive neuromodulation methods on key nodes of neural circuits. Created with BioGDP.com (65). Node colors represent different functional categories: blue = peripheral nerve regulation, green = neurotransmitter, purple = central nerve regulation. Ach, Acetylcholine; DLPFC, Dorsolateral Prefrontal Cortex; Hypo, Hypothalamus; Hipp, Hippocampus; LS, Limbic System; MNS, Median Nerve Stimulation; NE, Norepinephrine; 5-HT, Serotonin; OX, Orexin; DA, Dopamine; PCUN, Precuneus; AG, Angular Gyrus; RN, Raphe Nucleus; LC, Locus Coeruleus; SNc, Substantia Nigra pars compacta; TMS, Transcranial Magnetic Stimulation; tDCS, Transcranial Direct Current Stimulation; tACS, Transcranial Alternating Current Stimulation; TI, Temporal Interference; LITUS, Low-Intensity Transcranial Focused Ultrasound; TNS, Trigeminal Nerve Stimulation; taVNS, Transcutaneous Auricular Vagus Nerve Stimulation; Thal, Thalamus; ARAS, Ascending Reticular Activating System.

In 2023, Xu et al. (60) conducted an animal experiment to support the mechanism: 40-Hz TNS (0.2 mA, 200 μs, 60 min per day) was applied to TBI mice. The results showed that: (1) The mice’s working memory, novel object recognition, and olfactory associative learning abilities were significantly improved; (2) The pathological damage in the hippocampus was reversed; (3) TNS downregulated inflammatory factors (caspase-1) and injury-related genes (Cav2), and upregulated neuroprotective genes (brevican) and cognition-related genes through three neural circuits (TG → hypothalamic PVN CRH + neurons → SNc/VTA DAT + neurons → hippocampus). TNS has both neuroprotective and wake-promoting effects, diverse mechanisms of action, and great potential for future development (Figure 1).

## 5 Combined treatment regimens based on multi-circuit synergy

The regulatory effect of a single neural circuit is limited. Combining non-invasive neuromodulation techniques with different mechanisms of action can achieve multi-circuit synergistic repair and enhance treatment efficacy. Currently, there are two main types of combined regimens.

### 5.1 TMS + MNS combined therapy

#### 5.1.1 Circuit synergistic mechanism

TMS targets the frontoparietal network to enhance central neural circuit function by inducing synaptic remodeling and improving brain metabolism, while MNS activates the ARAS to dilate cerebral blood vessels, increase cerebral blood flow, optimize the intracerebral microenvironment, relieve neural cell inhibition, and promote synaptic connection reconstruction; the combination thus forms a synergistic effect of central circuit regulation plus peripheral arousal support (61).

#### 5.1.2 Clinical evidence

Xiong et al. (62) found that MNS combined with rTMS resulted in significantly better improvements in conscious level and neurological function than MNS or rTMS alone. The parameters included 10-Hz rTMS targeting the left DLPFC (90% RMT, 2,000 pulses per session, once daily) and MNS (20 mA, 300 μs, 40 Hz, 8 h daily), administered simultaneously for 4 weeks (62). Although all three groups (combined stimulation group, sham rTMS group, sham MNS group) showed improved behavioral assessment scores after treatment, the total CRS-R and GCS scores in the MNS + rTMS group were significantly higher, with wakefulness rates of 48% (rTMS group), 52% (MNS group), and 65% (combined group)—confirming the synergistic advantage.

A study (63) from the First Affiliated Hospital of Nanchang University included DOC patients of different age groups (young, middle-aged, elderly). After 4 weeks of combined rTMS and MNS treatment, the CRS-R, GCS, and Full Outline of UnResponsiveness (FOUR) scores of patients in all groups were significantly increased. Patients of all age groups benefited from the combined treatment, indicating a wide applicable population.

### 5.2 LITUS+TMS combined therapy

#### 5.2.1 Circuit synergistic mechanism

LITUS precisely regulates the cortico-thalamocortical circuit and features bidirectional regulation and deep targeting advantages.

TMS modulates the frontoparietal network. Synchronous application of LITUS and TMS may induce sustained cortical inhibition lasting up to 30 min, with a clear cumulative effect. MEP suppression during stimulation sessions 11–20 is significantly stronger than that observed during sessions 1–10, supporting long-term neuromodulation in DOC patients with multi-circuit dysfunction. Synchronous application entails triggering TMS 10 ms before the end of each LITUS pulse, with LITUS parameters set at 650 kHz frequency, 0.3 W/cm^2^ intensity, and 30-min duration per session, and TMS parameters set at 10 Hz, 90% RMT, 100 pulses per session (64). This synergistic post-inhibitory effect is strictly condition-dependent and occurs only when LITUS and TMS are delivered synchronously, with TMS triggered 10 ms before the end of LITUS, and when effective online cortical inhibition—defined as a real-time reduction in corticospinal excitability during stimulation—is achieved, thereby minimizing interference from non-target brain regions and enhancing stimulation specificity (64).

#### 5.2.2 Clinical evidence

Studies have confirmed that the inhibitory effect of LITUS alone on the motor cortex is only short-term (during and immediately after stimulation); TMS alone is difficult to produce a lasting post-effect (64).

Combined stimulation (especially synchronous stimulation LITUS-TMS-C) may induce a sustained inhibitory effect in consciousness-related brain regions (e.g., OI + populations) (64). This extendable and cumulative synergistic effect provides the possibility of more lasting functional improvement in consciousness-related brain regions.

LITUS alone, TMS alone, or separated TUS-TMS stimulation (TMS applied 2.5 s after the end of TUS) cannot induce this effect—confirming the specificity of combined stimulation (64).

### 5.3 Challenges of combined therapy

The synergistic effect of combined therapy has been initially confirmed, yet this treatment regimen still has multiple limitations: first of all, the relevant studies not only have a small sample size but also lack verification by multi-center randomized controlled trials (RCTs) (62); secondly, the optimal parameter combination in treatment, such as key elements including stimulation intensity and treatment interval, has not been clarified; in addition, the impact of different degrees of circuit damage on therapeutic efficacy has not been quantified from an anatomical perspective so far, which makes it impossible to clearly define the differences in efficacy among patients with different degrees of damage, and further research and optimization of the treatment regimen are urgently needed (63).

## 6 Summary and outlook

### 6.1 Outlook

Non-invasive neuromodulation technology has become an important direction for DOC treatment. Different techniques achieve consciousness improvement by targeting specific consciousness-related neural circuits: TMS, tDCS, MNS, and TMS + MNS combined therapy have the most sufficient clinical evidence, while deep regulation techniques such as LITUS and TI show great potential. Non-invasive neuromodulation technology target loops and advantages are summarized in Table 2.

**Table 2:** Summary of target loops and advantages of non-invasive neuromodulation techniques.

Treatment methods	Core targeting loop	Core advantage (*: based on clear mechanism of action)	Effective parameters	Ineffective/suboptimal parameters	Citations
TMS	Frontoparietal network	*The curative effect is clear, the target is clear, and the application is diverse; *regulates neuronal excitability and frontoparietal connectivity through electromagnetic induction	10–20 Hz, 90–100% RMT, 2,000–3,000 pulses/session, 10–14 consecutive days	5 Hz, <80% RMT, <5 days of stimulation	(23, 24, 26)
tDCS	Frontoparietal network	*There are many studies and applications, and the improvement of MCS patients is significant; *modulates cortical excitability through direct current-induced depolarization/hyperpolarization	2 mA, 20 min/session, 20 sessions total (twice daily for 10 days)	1 mA, <10 sessions, sham stimulation parameters	(28)
tACS	Cortex-thalamus − cortical loops	*It can oscillate synchronously with low adverse reactions	γ-band (35–140 Hz), right DLPFC + frontopolar cortex	Anti-phase stimulation, <10 Hz frequency	(31)
TI	Cortex-thalamus − cortical loops	*It generates an envelope electric field with high selectivity and great potential for regulation in deep brain regions; *avoids superficial cortex activation (high safety)	5 Hz envelope frequency, 2 mA intensity, 20 min/session	Single-frequency stimulation, >3 mA intensity (risk of side effects)	(41, 44)
LITUS	Cortex-thalamus − cortical loops	*Accurate and deep targeted stimulation to improve the neuronal microenvironment; *activates thalamic neurons and inhibits neuroinflammation	650 kHz, 0.3 W/cm^2^, 30 min/session, 5–10 consecutive days	>0.5 W/cm^2^ (risk of tissue damage), <15 min/session	(40, 43)
MNS	ARAS	*Activating the uplink network system to improve cerebral blood flow is applicable without etiologic doc; *promotes NE/5-HT release and orexin-A expression	20 mA, 300 μs, 40 Hz, 8 h/day, 2 weeks	<15 mA, <4 h/day stimulation duration	(5, 51)
taVNS	ARAS + LS	*Regulate the limbic system and affect the release of vagal neurotransmitters; *promotes NE/5-HT release and downregulates neuroinflammation	4 mA-6 mA, bilateral concha stimulation, 4 weeks	<3 mA, unilateral stimulation, <2 weeks	(48)
TNS	ARAS + LS	*Improve the release of neurotransmitters and regulate the microenvironment of brain regions	0.2 mA, 200 μs, 40 Hz, 60 min/day, 4 weeks (58)	>0.3 mA, <30 min/day stimulation duration	(58)
TMS + MNS	Frontoparietal lobe + ARAS	Synergetic enhancement of wake-promoting effect	10-Hz rTMS (left DLPFC) + MNS (20 mA, 40 Hz), 4 weeks	Asynchronous stimulation, <1 week of combined treatment	(65)
LITUS+TMS	Cortex-thalamus− cortex + Frontoparietal lobe	Synergism produces long- lasting and cumulative effects with strong specificity	LITUS (650 kHz, 0.3 W/cm^2^) + TMS (10 Hz, 90% RMT), TMS triggered 10 ms before LITUS end	TMS triggered >2.5 s after LITUS end, LITUS intensity >0.5 W/cm^2^	(62)

DLPFC, Dorsolateral Prefrontal Cortex; RMT, Resting Motor Threshold; Thal, Thalamus; ARAS, Ascending Reticular Activating System; LITUS, Low-intensity Transcranial Focused Ultrasound.

Future research should focus on three aspects. Several pivotal research directions should be prioritized to advance the development of combined therapy for disorders of consciousness (DOC), including clarifying circuit damage characteristics by identifying the differences in circuit damage among different DOC subtypes such as vegetative state/unresponsive wakefulness syndrome (VS/UWS) and minimally conscious state (MCS) to formulate personalized targeted treatment plans, optimizing treatment regimens through conducting large-sample, multi-center randomized controlled trials (RCTs) so as to refine stimulation parameters (e.g., frequency, intensity) and combined therapy protocols, and exploring regulatory mechanisms by carrying out in-depth studies on the molecular, cellular, and circuit-level mechanisms of non-invasive neuromodulation, which can provide solid theoretical support for precise treatment.

With the continuous development of technology, non-invasive neuromodulation is expected to achieve precise and efficient regulation of consciousness-related neural circuits, bringing rehabilitation hope to more DOC patients.
